# Impact of pharmacist-led antibiotic stewardship interventions on compliance with surgical antibiotic prophylaxis in obstetric and gynecologic surgeries in Nigeria

**DOI:** 10.1371/journal.pone.0213395

**Published:** 2019-03-07

**Authors:** Usman Abubakar, Syed Azhar Syed Sulaiman, Adebiyi Gbadebo Adesiyun

**Affiliations:** 1 Pharmacy Department, Ibrahim Badamasi Babangida Specialist Hospital, Minna, Nigeria; 2 Department of clinical Pharmacy, School of Pharmaceutical Sciences, Universiti Sains Malaysia, Penang, Malaysia; 3 Department of Obstetrics and Gynecology, Ahmadu Bello University Teaching Hospital, Zaria, Nigeria; University of North Dakota, UNITED STATES

## Abstract

**Background:**

Inappropriate and excessive use of surgical antibiotic prophylaxis are associated with the emergence of antibiotic resistance. Antibiotic prophylaxis malpractices are common in obstetrics and gynecology settings and antibiotic stewardship is used to correct such malpractice.

**Objective:**

To evaluate the impact of antibiotic stewardship interventions on compliance with surgical antibiotic prophylaxis practice in obstetrics and gynecology surgeries.

**Method:**

A prospective pre- and post-intervention study was conducted in two tertiary hospitals between May and December 2016. The duration of the each period was 3 months. Antibiotic stewardship interventions including development of a protocol, educational meeting and audit and feedback were implemented. Data were collected using the patient records and analyzed with SPSS version 23.

**Results:**

A total of 226 and 238 surgical procedures were included in the pre- and post-intervention periods respectively. Age, length of stay and estimated blood loss were similar between the two groups. However, specialty and surgical procedures varied significantly. There was a significant increase in compliance with timing (from 14.2% to 43.3%) and duration (from 0% to 21.8%) of surgical antibiotic prophylaxis after the interventions. The interventions significantly reduced the prescription of third generation cephalosporin (-8.6%), redundant antibiotic (-19.1%), antibiotic utilization (-3.8 DDD/procedure) and cost of antibiotic prophylaxis (-$4.2/procedure). There was no significant difference in the rate of surgical site infection between the two periods. Post-intervention group (OR: 5.60; 95% CI: 3.31–9.47), elective surgery (OR: 4.62; 95% CI: 2.51–8.47) and hospital attended (OR: 9.89; 95% CI: 5.66–17.26) were significant predictors of compliance with timing while elective surgery (OR: 12.49; 95% CI: 2.85–54.71) and compliance with timing (OR: 58.55; 95% CI: 12.66–270.75) were significantly associated with compliance to duration of surgical antibiotic prophylaxis.

**Conclusion:**

The interventions improve compliance with surgical antibiotic prophylaxis and reduce antibiotic utilization and cost. However, there is opportunity for further improvement, particularly in non-elective surgical procedures.

## Introduction

Surgical Site Infection (SSI) is one of the most common healthcare associated infection in developing countries [1]. In Nigeria, SSI accounts for 30.7% of all healthcare acquired infections and is ranked second after urinary tract infection [2]. Evidence show that the prevalence of SSIs in Nigeria is between 9.1% and 30.1% [3–5]with higher rate observed in obstetrics and gynecologic surgeries [2]. SSIs are associated with morbidity, mortality and healthcare cost. Patients who develop SSIs are two times more likely to die and they have 6 times higher risk of readmission into the hospital compared to those without SSI [6]. SSI also doubles the risk of admission into the intensive care unit and prolongs the duration of hospitalization [6]. The health care expenditure for patient who develop SSI is two times higher than those without SSIs [7].

Evidence demonstrate that surgical antibiotic prophylaxis; defined as the administration of antibiotic prior to incision with the ultimate goal of preventing infection after surgery, significantly reduce the incidence of SSIs [8, 9]. However, the efficacy of surgical antibiotic prophylaxis depends on the following factors: choice of antibiotic, time of administration, dose, re-dosing (in prolong surgery) and duration of administration [10]. Incorrect selection of antibiotic and suboptimal dose and timing diminish the efficacy of surgical antibiotic prophylaxis and increase the risk of SSIs while prolong use of prophylaxis is associated with emergence of antibiotic resistance [11, 12]. Surgical antibiotic prophylaxis practice among obstetricians and gynecologists in Nigeria is not compliant with guidelines [13]. Use of broader spectrum antibiotics, unnecessary combination of antibiotics, suboptimal timing and long duration of surgical antibiotic prophylaxis has been reported [13]. These malpractices have the potential to trigger the emergence and spread of antibiotic resistance. Available evidence shows that most Methicillin Resistant *Staphylococcus aureus* isolates in Nigeria are detected from surgical wound specimens [14].

Antibiotic stewardship is an antimicrobial resistance containment strategy aim at reducing inappropriate use of antibiotics for empirical, prophylactic and therapeutic purposes [15–18]. The main goals of antibiotic stewardship are to improve patients’ clinical outcomes and minimize adverse effects and antibiotic resistance [15–18]. The secondary goal is to reduce health care cost [15, 17, 19]. Several antibiotic stewardship interventions including: local antibiotic prophylaxis protocol/guideline [20]; educational training for prescribers (surgeons) [21]; audit and feedback [22] and some restrictive interventions such as automatic stop order and personalized antibiotic prophylaxis set [23] effectively improve compliance with surgical antibiotic prophylaxis [24–26]; optimize patients’ clinical outcomes [24, 25, 27, 28] and reduce cost of antibiotic prophylaxis [29] and antibiotic resistance [30]. To the best of our knowledge, there is no evidence to demonstrate the effectiveness of antibiotic stewardship interventions to correct surgical antibiotic prophylaxis malpractices in Nigeria. This study evaluates the impact of antibiotic stewardship interventions on prescribing (compliance choice, timing and duration of antibiotic prophylaxis and antibiotic utilization), clinical (SSIs rate) and economic (costs of antibiotic prophylaxis) outcomes.

### Ethical approval

Ethical approval was obtained from Ahmadu Bello University Teaching Hospital Health Research and Ethics Committee (reference number: ABUTH/HREC/T26/2016) and Aminu Kano Teaching Hospital Research Ethics Committee (reference number: AKTH/MAC/SUB/12A/P-3/VI/1735). Furthermore, verbal consent was taken from the participants prior to data collection. High level of confidentiality and anonymity was maintained throughout the study.

## Methods

### Study design, settings and sampling

This was a pre- and post-intervention study conducted in the department of obstetrics and gynecology in two public tertiary hospitals located in Northern Nigeria; Ahmadu Bello University Teaching Hospital (ABUTH), Zaria, and Aminu Kano Teaching Hospital (AKTH), Kano. Sample size was calculated using Pocok’s formula [31] and the following assumptions; overall risk of SSI before the intervention of 20.3% [32] and an estimated achievable decrease in SSI rate to 10.0% after intervention. With a significance level of 5% and a power of 80%, the require sample size was calculated as 218 procedures each before and after the intervention.

### Study population and inclusion criteria

Women who had elective and emergency obstetric and gynecologic surgeries were considered for inclusion. Surgeries with clean, clean-contaminated and contaminated wounds were selected. All adult patients were eligible to participate in the study. Cancer and Human Immunodeficiency Virus (HIV) seropositive patients, who have high risk of infection, and those with existing infection before incision were excluded from the study.

### Study period

The study was conducted in 3 phases: the pre-intervention (baseline), intervention and post intervention phase. The baseline was conducted between May 2016 and July 2016. Antibiotic stewardship interventions (development of protocol, education and audit and feedback) were implemented in August and September 2016 while the post-intervention evaluation was carried out between October 2016 and December 2016.

### Data collection

Data were collected using the patients’ medical, anesthesia, nursing and medication records. Information retrieved from the patient records include: dates of admission, surgery and discharge, patient’s age, type of surgery (elective or emergency), surgical procedure performed and estimated blood loss. Information regarding prescribed antibiotic(s) including: name of antibiotic(s), dose(s), time of administration of first dose (either at induction of anesthesia or post-operation) and duration of antibiotic prophylaxis was also documented. Resident doctors inspected patient’s wound during hospital stay and before they were discharged from the hospital. The outcome of wound inspection was also recorded.

### Antibiotic stewardship interventions

A bundle of antibiotic stewardship interventions was implemented after the pre-intervention period. The goal of the interventions was to reduce errors associated with surgical antibiotic prophylaxis. The interventions were targeted at changing the behavior of obstetricians and gynecologists regarding surgical antibiotic prophylaxis practice. The interventions include: development and dissemination of departmental protocol for surgical antibiotic prophylaxis; educational meeting with the obstetricians and gynecologists; audit and feedbacks using baseline data and reminder in the form of wall mounted posters. In both hospital, the protocol was developed by a team that comprise of 4–5 consultant obstetricians and gynecologists and a clinical pharmacist. The protocol was presented to the department by the clinical pharmacist before adoption. The protocol states that antibiotic prophylaxis in clean, clean-contaminated and contaminated wounds should be administered within 60 minutes before incision and discontinued within 24 hours after completion of surgery. The protocol recommends intravenous ampicillin-cloxacillin plus intravenous metronidazole (Hospital A) and intravenous amoxicillin-clavulanic acid (Hospital B) as the antibiotics of choice for surgical prophylaxis. The selection of amoxicillin-clavulanic acid in hospital B was due to high incidence of Extended Spectrum Beta-Lactamase (ESBL) infections [33]. The second intervention, educational meeting, was organized during the department’s clinical meeting and focus on the principles of surgical antibiotic prophylaxis for obstetrics and gynecology surgeries. The presentation was delivered by a clinical pharmacist. The data collected during the pre-intervention period was presented to the clinicians and areas where practice did not align with guidelines were highlighted.

### Outcome measures

#### (a) Prescribing outcomes

The prescribing outcomes evaluated were the compliance with timing and duration of antibiotic prophylaxis. Compliance with timing was defined as administration of an intravenous antibiotic within 60 minutes before incision while compliance with duration of surgical antibiotic prophylaxis was defined as use of a single dose of antibiotic or discontinuation of antibiotic prophylaxis within 24 hours after completion of surgery. High rate of redundancy in surgical antibiotic prophylaxis has been reported in the study settings [13]. Therefore, the impact of the interventions on unnecessary antibiotic combination was measured. Redundancy was defined as the prescription of two antibiotics with overlapping spectra of activity for surgical antibiotic prophylaxis and which lack evidence to demonstrate synergy [34].

#### (b) Antibiotic utilization

Antibiotic consumption was calculated using the Anatomical Therapeutic Chemical/Defined Daily Dose (ATC/DDD) index (2017) of the World Health Organization (WHO) Collaborating Centre for Drugs Statistics Methodology [35]. Defined Daily Dose was defined as the assumed average maintenance dose per day of an antibiotic used for its main indication in an adult [35].

#### (c) Clinical outcome

SSI was defined using the Centre for Disease Control and Prevention–National Healthcare Safety Network (CDC-NHNS) criteria [36]. However, the surveillance of SSI in this study was limited to the period of hospitalization.

#### (d) Economic outcome

Cost of surgical antibiotic prophylaxis was calculated using the price list of medication obtained from the pharmacy department in each hospital. The unit cost of each dose was multiplied by the total number of doses. The lowest price for generic antibiotics on the price list was used to estimate the costs of antibiotic prophylaxis.

### Data analysis

Data were entered, coded, cleaned and analyzed using Statistical Package for the Social Sciences (SPSS) version 23. All categorical variables were presented in frequency and percentage while continuous variables were expressed as mean and standard deviation. Pearson Chi Square Test was used to compare patient’s demographic and surgical characteristics between the pre- and post-intervention groups. Continuous variables during the two periods were compared using independent T-test. Compliance with antibiotic selection, timing and duration of antibiotic prophylaxis and rate of SSI during the pre- and post-intervention period were compared using Pearson Chi square and Fisher exact (where appropriate) Tests. Antibiotic utilization and cost of antibiotic prophylaxis in the pre- and post-intervention period were compared using T-test or Mann-Whitney U Test. Logistic regression analysis was used to determine the predictors of compliance with timing and duration of surgical antibiotic prophylaxis. Data was de-identified before analyses and the names of the hospitals were recoded as “Hospital A” and “Hospital B.” P value < 0.05 was considered to be statistically significant in all the inferential statistical analyses.

## Results

Both hospitals had a team that comprise of Consultants, Senior Registrar, Registrar and House officers who provided obstetrics and gynecology care to the patients. There was an infection control team in each hospital. Table 1 shows the characteristics of the hospitals that participated in the study.

**Table 1:** Characteristics of the hospitals.

Characteristics	Number	
	Hospital A	Hospital B
Total bed-size	547	500
Bed-size in the department of obstetrics and gynecology	91	93
Physician cadre		
Consultants OBG	17	13
Senior registrar	17	14
Registrar	20	14
House officers	25–30	8–12
Presence of infection control team	Yes	Yes

### Comparison of patient and surgical characteristics between the pre- and post-intervention periods

There were 226 and 238 surgical procedures in the pre- and post-intervention periods respectively. No significant difference in the mean age, length of hospital stay and estimated blood loss was observed between the two periods. However, elective surgeries and gynecology procedures were significantly higher during the post-intervention period. Table 2 demonstrates the comparison of the patient’s and surgical characteristics between the pre- and post-intervention periods.

**Table 2:** Comparison of patients’ demographic and surgical characteristics between the pre- and post-intervention period.

Variables	Number of surgical procedures (%)		P value
	Pre-intervention (n = 226)	Post-intervention (n = 238)	
**Hospital**			0.529
Hospital A	67 (29.6)	77 (32.4)	
Hospital B	159 (70.4)	161 (67.6)	
**Specialty**			0.006
Obstetrics	176 (77.9)	158 (66.4)	
Gynecology	50 (22.1)	80 (33.6)	
**Type of surgery**			0.014
Elective	113 (50.0)	146 (61.3)	
Emergency	113 (50.0)	92 (38.7)	
**Surgical procedure**			0.009
Caesarean section	175 (77.4)	158 (66.4)	
Myomectomy	17 (7.5)	23 (9.7)	
Hysterectomy	11 (4.9)	17 (7.1)	
Laparotomy	13 (5.8)	11 (4.6)	
Laparoscopy	5 (2.2)	16 (6.7)	
Others	5 (2.2)	13 (5.5)	
Mean age (years)	31.9 ± 8.0	31.2 ± 7.4	0.338
Mean Length of stay(days)	6.4 ± 2.8	6.1 ± 2.6	0.288
Mean Estimated Blood Loss (mls)	390 ± 168	409 ± 173	0.266

Pearson Chi-Square

### Prescribing outcomes

#### Impact of antibiotic stewardship interventions on compliance with timing and duration of surgical antibiotic prophylaxis

Overall, compliance with timing of surgical antibiotic prophylaxis was increased from 14.2% in the pre-intervention period to 43.3% in the post-intervention period (P < 0.001). Compliance with timing was significantly increased in both hospitals and surgical specialties. No difference in compliance with timing of administration was observed in emergency surgeries after the interventions. Compliance with timing was significantly improved in elective surgeries from 10.6% to 58.9%; P < 0.001 (Table 3). Compliance with duration of surgical antibiotic prophylaxis was also improved after the interventions from 0% to 21.8% (P < 0.001). Increase in compliance with duration of surgical antibiotic prophylaxis was observed in both hospitals, both surgical specialties and in elective surgeries (Table 3).

**Table 3:** Comparison of compliance with timing and duration of surgical antibiotic prophylaxis between the pre- and post-intervention periods.

Variable	Number of surgical procedures that received antibiotic prophylaxis within 60 minutes before incision (%)		P value	Number of surgical procedures with antibiotic prophylaxis discontinued within 24 hours after surgery (%)		P value
	Pre intervention	Post intervention		Pre intervention	Post intervention	
**Hospital**						
Hospital A	27 (34.6)	51 (65.4)	0.002	0 (0.0)	19 (24.7)	0.001
Hospital B	5 (3.1)	52 (32.3)	< 0.001	0 (0.0)	33 (20.3)	< 0.001
Overall	32 (14.2)	103 (43.3)	< 0.001	0 (0.0)	52 (21.8)	< 0.001
**Specialty**						
Obstetrics	22 (12.5)	60 (38.0)	< 0.001	0 (0.0)	36 (22.8)	< 0.001
Gynecology	10 (20.0)	43 (53.8)	< 0.001	0 (0.0)	16 (20.0)	0.001
**Type of surgery**						
Elective	12 (10.6)	86 (58.9)	< 0.001	0 (0.0)	49 (33.6)	< 0.001
Emergency	20 (17.7)	17 (18.5)	0.885	0 (0.0)	3 (3.3)	0.889
**Surgical procedure**						
Caesarean section	23 (13.3)	60 (38.0)	< 0.001	0 (0.0)	36 (22.8)	< 0.001
Myomectomy	1 (5.9)	13 (56.5)	0.001	0 (0.0)	2 (8.7)	0.499
Hysterectomy	4 (36.4)	10 (58.8)	0.246	0 (0.0)	0 (0.0)	1.000
**Laparotomy	3 (23.1)	0 (0.0)	0.223	0 (0.0)	0 (0.0)	1.000
**Laparoscopy	1 (20.0)	13 (81.3)	0.025	0 (0.0)	11 (68.8)	0.012
**Others	0 (0.0)	7 (53.8)	0.101	0 (0.0)	3 (23.1)	0.522

Pearson Chi-Square

**Fisher Exact test

#### Impact of antibiotic stewardship interventions on antibiotic selection and redundancy

Prescription of third generation cephalosporin for surgical antibiotic prophylaxis was reduced from 29.2% in the pre-intervention period to 20.6% in the post-intervention period (P = 0.032). Overall, the rate of redundant antibiotic prescription was reduced by 19.1% (from 70.8% in the pre-intervention period to 51.7% in the post-intervention period; P < 0.001). Combination of antibiotic with redundant spectra of activity was eliminated in Hospital A and was reduced significantly in Hospital B (from 99.4% to 76.4%; P < 0.001) during the post-intervention period.

### Impact of antibiotic stewardship interventions on antibiotic utilization and cost of surgical antibiotic prophylaxis

There was a significant decrease in antibiotic utilization after the interventions. The DDD of surgical antibiotic prophylaxis declined from 16.6 ± 3.6 DDD/procedure in the pre-intervention period to 12.8 ± 6.8 DDD/procedure in the post-intervention period (P < 0.001). Antibiotic utilization was reduced from 13.2 ± 4.6 DDD/procedure to 10.6 ± 5.5 DDD/procedure in Hospital A (P = 0.003) and from 18.1 ± 1.6 DDD/procedure to 13.9 DDD/procedure in Hospital B (P < 0.001) (see Table 4). The mean cost of surgical antibiotic prophylaxis was reduced by $4.2 (P < 0.001) after the interventions (Table 4).

**Table 4:** Comparison of antibiotic utilization and cost of surgical antibiotic prophylaxis between the pre- and post-intervention period.

Variable	Mean DDD/procedure (SD)		P value	Mean cost of antibiotic prophylaxis (USD)		
	Pre-intervention	Post-intervention		Pre-intervention	Post-intervention	P value
**Hospital**						
Hospital A	13.2 ± 4.6	10.6 ± 5.5	0.003	13.3 ± 3.6	7.6 ± 4.8	< 0.001
Hospital B	18.1 ± 1.6	13.9 ± 7.1	< 0.001	17.4 ± 2.1	14.0 ± 6.1	< 0.001
Overall	16.6 ± 3.6	12.8 ± 6.8	< 0.001	16.2 ± 3.2	12.0 ± 6.5	< 0.001

Independent Student T test

### Predictors of compliance with timing and duration of surgical antibiotic prophylaxis

Multivariate logistic regression analysis shows that antibiotic stewardship interventions increased compliance with timing of surgical antibiotic prophylaxis. Procedures in the post-intervention period were 5.6 times more likely to receive surgical antibiotic prophylaxis within 60 minutes before incision (95% CI: 3.31–9.47; P < 0.001). Elective surgery (OR: 4.62; 95% CI: 2.51–8.47; P < 0.001) and attendance at Hospital A (OR: 9.89; 95% CI: 5.66–17.26; P < 0.001) were also significant predictors of compliance with timing of surgical antibiotic prophylaxis. In addition, elective surgery (OR: 12.49; 95% CI; 2.85–54.71; P = 0.001) and compliance with timing (OR: 58.55; 95% CI: 12.66–270.75; P < 0.001) were significantly associated with compliance with duration of surgical antibiotic prophylaxis (Table 5).

**Table 5:** Multivariate logistic regression analysis for predictors of compliance with timing and duration of surgical antibiotic prophylaxis.

Variable	Predictors of compliance with timing			Predictors of compliance with duration		
	AOR	95% CI	P value	AOR	95% CI	P value
Intervention	5.60	3.31–9.47	< 0.001	123 x 10^5^	0.00 -	0.994
Elective surgery	4.62	2.51–8.47	< 0.001	12.49	2.85–54.71	0.001
Specialty (Obstetrics)	1.18	0.67–2.10	0.553	4.57	1.83–11.38	0.001
Hospital	9.89	5.66–17.26	< 0.001	0.80	0.32–2.00	0.634
Compliance with timing	-	-		58.55	12.66–270.75	< 0.001

AOR = Adjusted Odds Ratio

### Impact of stewardship interventions on clinical outcome

Surveillance during hospitalization revealed that there was no significant difference in the incidence of SSI between the two periods. The rate of SSI in the pre-intervention period was 4% compared to 3.4% in the post-intervention period (P = 0.722).

## Discussions

The current study evaluates the impact of antibiotic stewardship interventions on compliance with surgical antibiotic prophylaxis in two hospitals in Nigeria. The outcome shows that development of a protocol, educational meeting coupled with audit and feedback improved compliance with timing of surgical antibiotic prophylaxis. This observation was consistent with studies conducted in South Africa, Egypt and Korea [21, 37, 38]. In our study, although compliance with timing increased after the interventions, antibiotic was administered after incision in many surgical procedures, particularly in emergency cases. This implies that the interventions were more effective in elective than non-elective surgeries. This could be explained by the policy of the hospitals which requires the patient to purchase antibiotic before they are administered. In addition, one cannot rule out the resistance of the prescribers to change practice considering the pharmacist-led nature of the interventions. Some authors have argued that physicians are more likely to change practice when it is introduced by their colleagues [22, 39].

The current study also found that compliance with duration of surgical antibiotic prophylaxis was significantly increased by 21.8%. Our finding was in consonance with a Chinese study which reported that pharmacist-led educational training for obstetricians coupled with prospective audits and feedbacks significantly increase compliance with duration of surgical antibiotic prophylaxis from 0.0% to 19.3% [25]. Also, an Egyptian study showed that education of clinicians, prospective audit and feedback and wall mounted posters significantly increased compliance with duration of surgical antibiotic prophylaxis in obstetric and gynecologic surgeries from 1.5% in the pre-intervention period to 37.5% in the post-intervention [21]. The current study demonstrates that the interventions improved use of antibiotics. However, the duration of surgical antibiotic prophylaxis was prolonged in more than two-third of the procedures in the post-intervention period. This could be attributed to the misperception among the prescribers that extended use of surgical antibiotic prophylaxis was associated with lower risk of SSI. In addition, some of the obstetricians and gynecologists believed that single dose or 24 hours surgical antibiotic prophylaxis was not applicable in Nigeria because of differences in terms of microbial contamination between hospitals in the country and those abroad. Similar sentiments have been reported in a previous study conducted in Africa [21].Another finding of our study was that the implementation of antibiotic stewardship interventions leads to a significant 8.6% reduction in the prescription of third generation cephalosporin for surgical antibiotic prophylaxis. Previous studies have reported that implementation of antibiotic stewardship program in urologic and obstetric surgeries was associated with reduction in the prescription of broad spectrum and expensive antibiotics [25, 40]. In addition to the reduction in the prescription of third generation cephalosporin, the current study observed a significant 19% decrease in the use of redundant anti-anaerobic antibiotics. This outcome was in agreement with a Korean study that demonstrated that clinician education coupled with prospective audits and feedbacks reduced the rate of unnecessary dual anti-anaerobic prescription from 42.3% before the intervention to 13.6% after the interventions [41]. The interventions were effective in reducing the rate of redundant antibiotic prescription in the current study, however, approximately 52% of the procedures in the post-intervention period had antibiotics with redundant spectra of activity. This implies that there is ample room for improvement to reduce inappropriate use of antibiotics in patients who undergo surgery. Future studies should consider implementing a more intensive prospective audit and feedback to reinforce the information and correct surgical antibiotic prophylaxis malpractices. Evidence has demonstrated that audit and feedbacks is more effective if it is provided more than once [22].

There was also a significant reduction (22.8%) in antibiotic utilization after the interventions in our study. This decline was attributed to decrease in the rate of redundancy and increase in compliance with duration of surgical antibiotic prophylaxis. Antibiotic stewardship interventions have been associated with 14% - 35% reduction in antibiotic utilization [24, 30, 42]. However, utilization of surgical antibiotic prophylaxis during the post-intervention period in our study (1279 DDD/100 procedures) was several folds higher than previous studies (79–129 DDD/100 procedures) [24, 30, 42]. This depicts overutilization of surgical antibiotic prophylaxis in our study settings and could trigger the emergence and spread of antibiotic resistance. More intensive approach is required to reduce the overutilization of surgical antibiotic prophylaxis. The current study also found that the implementation of antibiotic stewardship interventions led to a $4.2 decrease in the cost of surgical antibiotic prophylaxis per procedure. This represents a 26% reduction and was consistent with the result of a study conducted in the Netherlands [24]. Evidence in China showed that antibiotic stewardship program was associated with 71%– 95% decrease in cost of antibiotic prophylaxis per procedure and the cost benefit ratio range between $1:$11 and $1:$27 [25, 40, 43].

Our study discovered that increase in compliance with timing did not reduce the rate of SSI neither did reduction in the duration of antibiotic prophylaxis worsen the clinical outcome. The rate of SSI before and after the interventions were similar. However, it is important to note that surveillance of SSI was limited to the period of hospitalization. This limitation could explain the lack of significant difference between the two groups because evidence has shown that about 80% of all SSIs occur after a patient is discharged from the hospital [36]. The outcome of our study despite the limitation was in agreement with previous studies that have shown no significant reduction in the incidence of SSI after implementing antibiotic stewardship interventions [24, 27, 28, 44].

Multivariate logistic regression analysis demonstrated that procedures performed in the post-intervention period were 5.6 times more likely to receive surgical antibiotic prophylaxis within 60 minutes before incision compared to those carried out during the pre-intervention period. This was a confirmation that the improvement in timing could be attributed to the intervention and not confounding factors. In addition, elective surgical procedures and attendance at Hospital A were also significantly associated with compliance with timing and duration of surgical antibiotic prophylaxis. Compliance with timing was found to have a chain effect as it was associated with more than 58.5 times higher likelihood of compliance with duration of surgical antibiotic prophylaxis. Considering the fact that duration of surgical antibiotic prophylaxis was prolonged in all the procedures in the pre-intervention period, it could be argued that the improvement was a result of the interventions implemented.

The current study has some limitations including lack of randomization. However, quasi experimental design with a pre- and post-intervention is often used to determine the impact of healthcare interventions. The study was conducted in two tertiary hospitals and this would limit the generalizability of the result. Another limitation of this study was the incomplete surveillance for surgical site infections among the patients. This limits the reliability of the impact of the interventions on clinical outcome. In addition, other factors that affect SSI including: dose, re-dosing, comorbidities (diabetes mellitus, obesity, anaemia) and patients’ characteristics were not reported in the current study. The cost of surgical antibiotic prophylaxis was calculated based on the price of generic antibiotic irrespective of the brand administered. Therefore, the cost of antibiotic prophylaxis presented in the current study was an estimate and not the actual drug cost.

## Conclusions

Our study demonstrates that antibiotic stewardship interventions significantly improved compliance with timing and duration of surgical antibiotic prophylaxis and reduced redundancy, antibiotic utilization and cost without compromising clinical outcome. However, there is still room for improvement, particularly in emergency surgical procedures. The intervention, elective surgery and hospital attended were significant predictors of compliance with the timing and duration of surgical antibiotic prophylaxis.
